# Breaking the Immune Complexity of the Tumor Microenvironment Using Single-Cell Technologies

**DOI:** 10.3389/fgene.2022.867880

**Published:** 2022-05-16

**Authors:** Simone Caligola, Francesco De Sanctis, Stefania Canè, Stefano Ugel

**Affiliations:** Immunology Section, Department of Medicine, University of Verona, Verona, Italy

**Keywords:** immune system, single-cell technologies, cancer, tumor microenvironment, single-cell data analysis

## Abstract

Tumors are not a simple aggregate of transformed cells but rather a complicated ecosystem containing various components, including infiltrating immune cells, tumor-related stromal cells, endothelial cells, soluble factors, and extracellular matrix proteins. Profiling the immune contexture of this intricate framework is now mandatory to develop more effective cancer therapies and precise immunotherapeutic approaches by identifying exact targets or predictive biomarkers, respectively. Conventional technologies are limited in reaching this goal because they lack high resolution. Recent developments in single-cell technologies, such as single-cell RNA transcriptomics, mass cytometry, and multiparameter immunofluorescence, have revolutionized the cancer immunology field, capturing the heterogeneity of tumor-infiltrating immune cells and the dynamic complexity of tenets that regulate cell networks in the tumor microenvironment. In this review, we describe some of the current single-cell technologies and computational techniques applied for immune-profiling the cancer landscape and discuss future directions of how integrating multi-omics data can guide a new “precision oncology” advancement.

## Introduction

Cancer is one of the leading causes of death worldwide, accounting for nearly 10 million deaths in 2020 (https://www.who.int/news-room/fact-sheets/detail/cancer). Unfortunately, the pandemic COVID-19 will have consequences for cancer patients in coming years, since it has been associated with delays in diagnosis as well as interruption of therapeutic treatments and follow-up care. Hence the number of cancer victims will increase in the near future. Identifying cancer as a genetic disease characterized by a set of genomic aberrations, including in-frame insertions or deletions, missense amino acid changes, and large copy number variations, initially ingrained a “cancer cell-centric” vision in the scientific community where cancer cell-intrinsic properties exclusively drove tumorigenesis (Reddy et al., 1982; Santos et al., 1984; Lengauer et al., 1998; Futreal et al., 2004; Tomlins et al., 2005; Hanahan and Weinberg, 2011). Therefore, recognizing driver gene modifications has been a central aim of cancer research over the past 30 years, resulting in global initiatives such as The Cancer Genome Atlas (TCGA) (https://www.cancer.gov/about-nci/organization/ccg/research/structural-genomics/tcga) and the Catalog of Somatic Mutations in Cancer (COSMIC) (Tate et al., 2019) which include a broad collection of large-scale, systematic sequencing studies that constitute comprehensive catalogs of mutational abnormalities in the major tumor types. More than 100,000 somatic mutations in cancer genomes have been identified in the quarter-century since the first somatic mutation was reported (Zack et al., 2013), opening a new age for the classification and treatment protocols of some cancers, such as breast (Cancer Genome Atlas, 2012b; Ciriello et al., 2015), cervical (Cancer Genome Atlas Research Network, 2017), colorectal (Cancer Genome Atlas, 2012a), pancreatic (Consortium, 2020), gastric (Cancer Genome Atlas Research, 2014a), prostate (Cancer Genome Atlas Research, 2015), and lung carcinomas (Cancer Genome Atlas Research, 2012; Cancer Genome Atlas Research, 2014b).

In addition to the study of genetic diversity as a source of tumor cell heterogeneity, stable epigenetic changes in cancer have been received intense interest since they derive from poised or initiated chromatin states of several genes that can modulate expression of different pathways (Brown et al., 2014). Indeed, alterations of cellular activities, such as cell growth and differentiation, can be driven by epigenetic events that involve DNA methylation, histone modification, the readout of these modifications, chromatin remodeling and the effects of noncoding RNA (Feinberg et al., 2016). These alterations are temporary or yet long-lasting and they impact on tumorigenesis. For instance, aberrant DNA methylation has been, generally, associated with cancer development by inactivating gene transcription or repressing gene transcription and affecting chromatin structure (Baylin and Jones, 2011; Hanahan and Weinberg, 2011). Indeed, some gene promoters, especially key tumor suppressor genes, are unmethylated in normal tissues and highly methylated in cancer (Baylin and Jones, 2011). Interestingly, DNA methylation in cancer has generally been associated with tumor size (Feinberg et al., 2016) as well as with drug resistance and predicting response to treatment (Brown et al., 2014). Therefore, the comprehensive genetic and epigenetic analysis of cancer genomes has been for many years the most effective way to identify causative changes involved in tumorigenesis.

Despite the achievement of these extraordinary milestones in deciphering cancer cell biology, new insights demonstrate that aberrant genetic profiles of transforming cells alone are required but insufficient to nurse tumor development and progression. Indeed, cancer cells need to alter the stromal framework of the microenvironment to manipulate diverse physiological processes, such as promoting angiogenesis and vasculogenesis, to provide adequate nutrients and oxygen, and alter immune responses to avoid activating tumor-fighting elements, such as cytotoxic T lymphocytes (CTLs) (Hanahan and Weinberg, 2011). Therefore, “cancer-cell extrinsic” factors, such as local inflammation, metabolic switch, and immunity, are critical in fueling cancer growth. At this point, the key question is whether these extrinsic factors are independent of the genetic profile of cancer cells. A cornerstone study demonstrated a remarkable difference in the composition of tumor-infiltrating leukocytes in different tumor types by analyzing data on clinical outcomes and gene expression of 18,000 human tumors (Gentles et al., 2015). Interestingly, memory CD4^+^ T lymphocyte frequency correlated positively with a favorable outcome in lung cancer patients, whereas the same cell subset was associated with a worse outcome in patients affected with bladder tumors (Gentles et al., 2015), suggesting that cancer cell-intrinsic features can dictate the immune landscape of the tumor microenvironment (TME). Indeed, oncogene-driven modifications can alter tumor immunogenicity in a completely different way. Ongoing mutational processes generate either cancer neoantigens capable of activating tumor-eliminating immune cells (Balachandran et al., 2017; Luksza et al., 2017; Keenan et al., 2019) or produce immune soluble factors such as interleukins (e.g., IL-6, IL1-β), growth factors (e.g., granulocyte-macrophage colony-stimulating factor (GM-CSF)), and chemokines (e.g., CCL4), capable of differentiating immune cells into pro-tumor elements (Bronte et al., 2006; Pylayeva-Gupta et al., 2012; Calcinotto et al., 2018; Fiore et al., 2018; Vitale et al., 2019; Hofer et al., 2021). Host immune cells also influence cancer progression (Schreiber et al., 2011). The notion that tumors derived from immunodeficient hosts are more immunogenic than those derived from immunocompetent mice allowed us to hypothesize that the immune system actively shapes cancer cells, promoting the acquisition of genetic aberrations that can compromise cancer cell immunogenicity, favoring tumors escaping immune attacks. Several clinical and pre-clinical observations have validated the cancer immunoediting theory. For instance, two large studies demonstrated that tumor-infiltrating immune subsets in colorectal cancer were significant independent prognostic markers as well as microsatellite instability, long interspersed nucleotide element-1 (LINE-1) hypomethylation, and BRAF mutations (Ogino et al., 2009; Nosho et al., 2010). Moreover, the linear correlation between the density of effector memory CTLs at the site of the primary tumor and the survival of patients explicitly revealed the importance of TME immunity in cancer control (Galon et al., 2006; Fridman et al., 2017). Thus, the definition of the cancer genome and immune landscape of the TME is hierarchically equivalent and complementary to predict disease progression and therapeutic outcome.

**FIGURE 1:**
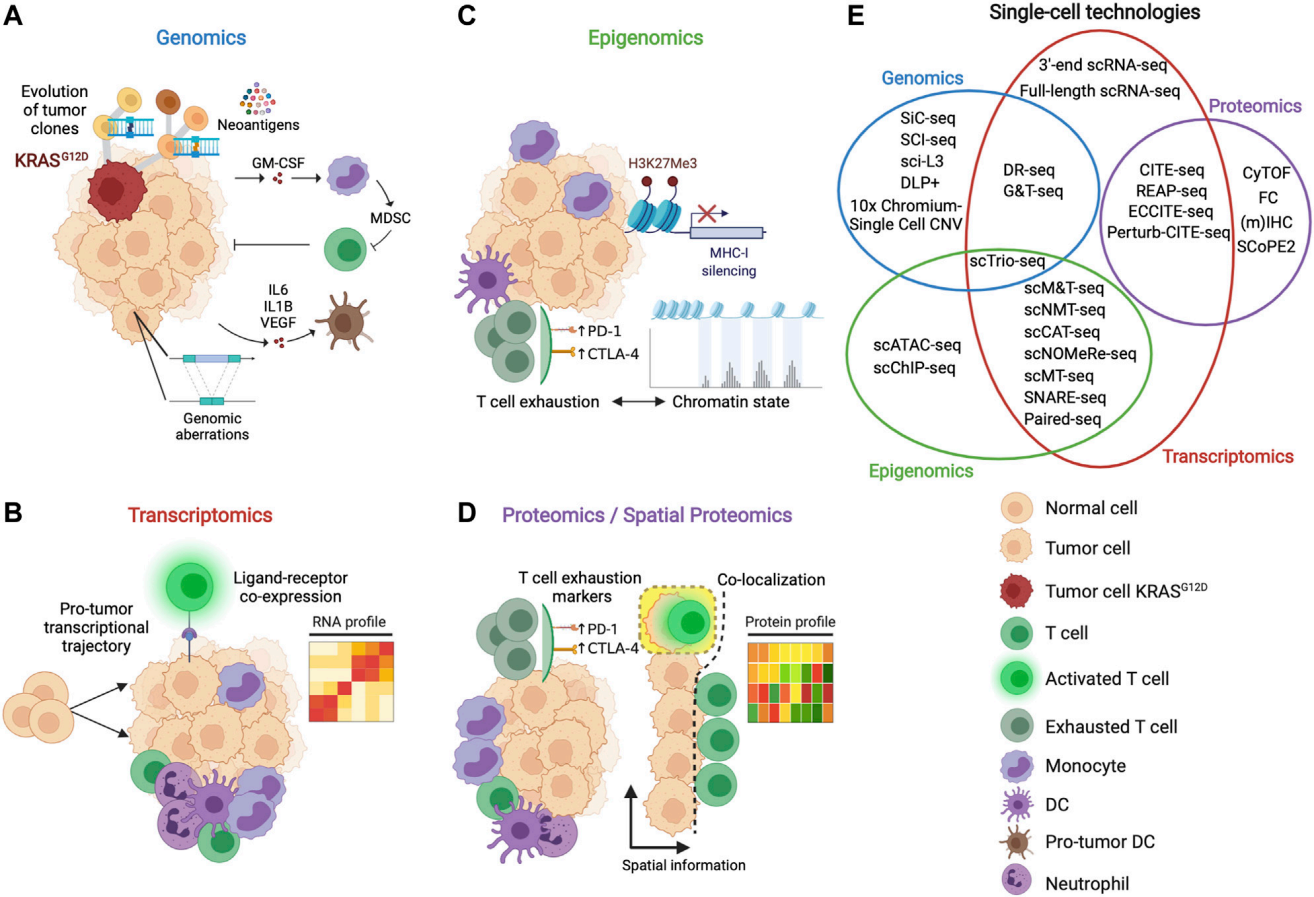
Multi-omic perspective to study the features of the TME. **(A)** Genomics analysis informs about how the tumor mutational landscape influences the TME, favoring, for example, the production of cytokines (e.g., IL-6, IL1-β) and growth factors (e.g., GM-CSF) inducing the proliferation of suppressive myeloid cells and the pro-tumor differentiation of antigen-presenting cells. **(B)** Transcriptomic analysis enables to inspect the transcriptional machinery of the single cells of the TME, deciphering, for example, developmental trajectories, cell states and cell-cell interactions. **(C)** Epigenomic analysis reveals how specific switches such as histone methylation and chromatin dynamics regulate different mechanisms capable to interfere with anti-tumor immune recognition and effector functions. **(D)** Proteomics provides information about the state of activation of the immune cells of the TME looking at the expression of immunomodulatory proteins such as checkpoint inhibitors (e.g., PD-L1, CTLA-4). Spatial proteomics gives additional information about the localization of the cells allowing, for example, to identify cell that interact in the TME. **(E)** Venn diagram depicting single-cell technologies to study single (non-overlapping sets) or multimodal (overlapping sets) omics. Genomics, transcriptomics, epigenomics and proteomics are represented as blue, red, green, and purple sets, respectively.

Immune cell heterogeneity and the presence of various cell subsets complicate TME immune profiling (Binnewies et al., 2018). Moreover, the spatial distribution of immunity within the tumor mass is a critical parameter that significantly influences immune cells acquiring pro- or anti-tumor functions (Bindea et al., 2013; Sautes-Fridman et al., 2019). A multi-omic perspective considering genomics, transcriptomics, epigenomics and proteomics is necessary to unveil the immune complexity of the TME (Figures 1A–D). Unfortunately, traditional technologies used to evaluate the immune landscape of the TME, such as immunohistochemistry (IHC), flow cytometry (FC), and bulk analysis of genomic, transcriptional, and proteomic analyses, have several limitations, such as large amount of biological material requirements, fewer parameters tested simultaneously, and low analysis resolution (Peregrin et al., 1973; Bendall et al., 2012; Abel et al., 2014). Recently, high-dimensional single-cell techniques (Finotello and Eduati, 2018; Chen G. et al., 2019; Gohil et al., 2021) have revolutionized the approach to decipher the cellular diversity, cell interactions and dynamics that exists in the TME and heterogeneity across patients by resolving cell subset complexity at the single-cell level using unsupervised clustering to identify potential unknown subpopulations of cells within the populations under study (Thorsson et al., 2019; Wagner et al., 2019; Zhao J. et al., 2020; Kieffer et al., 2020; Cheng et al., 2021). Here, we review how single-cell technologies (Figure 1E) and related computational techniques have improved our knowledge about the TME and discuss future applications of these cutting-edge techniques in immune-oncology to develop more effective personalized immunotherapy.

## Single-Cell Technologies to Study the TME

### Single-Cell Genomics

Several studies have demonstrated the connection between the tumor mutations and the immune composition or TME. Deciphering the complexity of the mutational landscape of tumor cell clones and sub-clones that underlies intratumor heterogeneity is necessary to understand tumor patient lethal outcomes, therapeutic failures, and drug resistance (McGranahan and Swanton, 2017). Single-cell genomics technologies can make an important contribution to this goal. However, the study of tumor clonality by analyzing single-nucleotide variations (SNVs) and copy number variations (CNVs) at the single-cell level is challenging. Most methods for creating single-cell libraries rely on whole-genome amplification (WGA) to overcome the inability of sequencing technologies to capture single-cell DNA molecules in low amounts of material. Since the first method based on degenerate oligonucleotide-primed PCR (DOP-PCR) (Telenius et al., 1992), several methods have been developed to increase the coverage and uniformity of the genome to allow both SNVs and CSVs to be studied within the same experiment (Dean et al., 2001; Zong et al., 2012), given the importance of the WGA step. More recent methods based on tagmentation (Adey et al., 2010), such as direct library preparation (DLP) (Zahn et al., 2017), and linear amplification via transposon insertion (LIANTI) (Chen et al., 2017), outperformed WGA methods in terms of accuracy, time, and cost, expanding potential future application scenarios.

**FIGURE 2:**
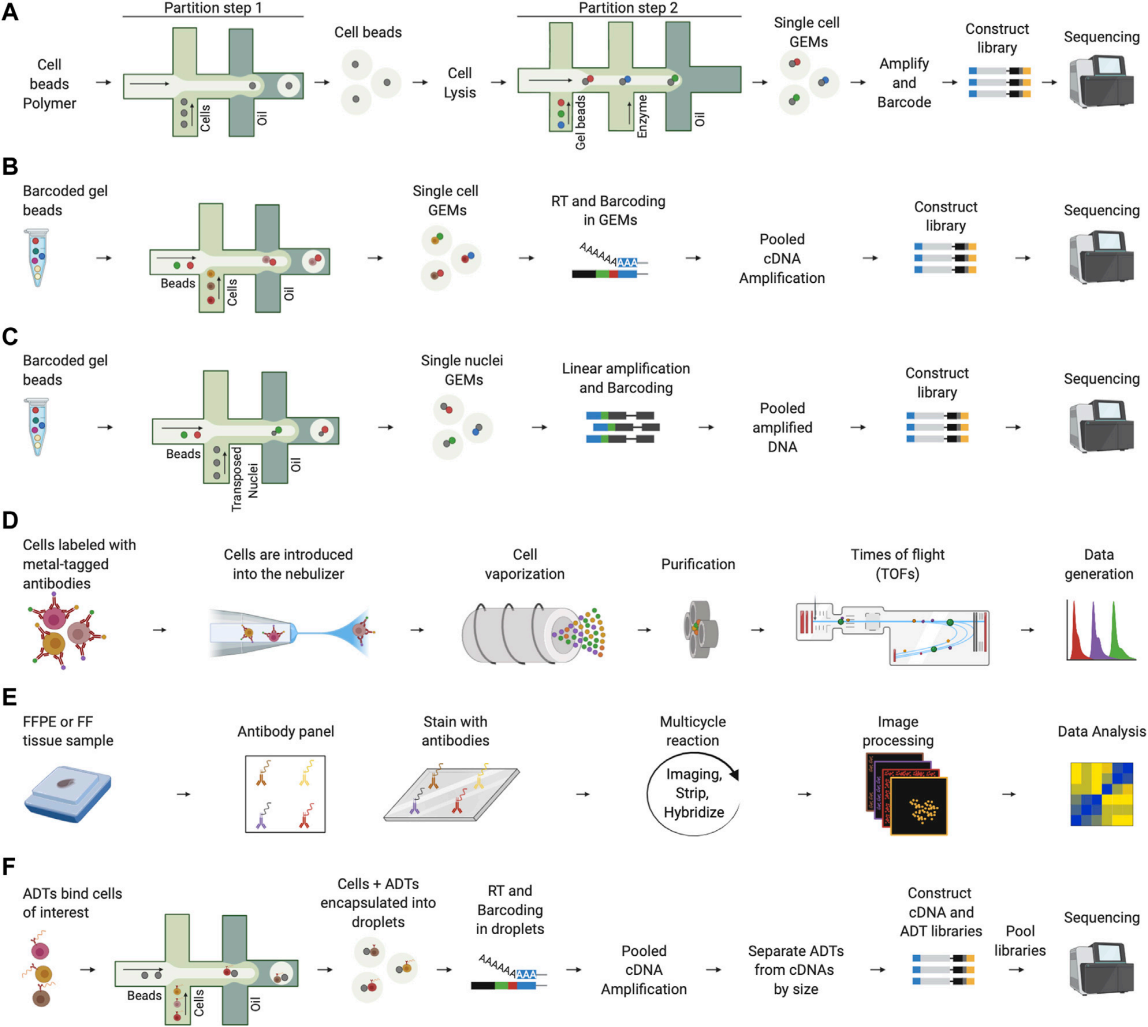
Representative single-cell technologies to study the TME. **(A)** 10x Genomics single-cell genomics involves two steps of encapsulation using the microfluidics system. In the first step, cells are partitioned using a cell beads polymer. The obtained cell beads are lysed to denature the genomic DNA and a second step on microfluidics chip is performed to encapsulate cell beads with barcode gel beads. After collecting single cell GEMs, amplification and barcoding of fragments is performed prior to breaking the emulsion and constructing the library for sequencing. **(B)** In 10x Genomics scRNA-seq, cells are encapsulated into droplets together with barcoded beads. Next, reverse transcription (RT) is performed in the collected GEMs and barcoded cDNAs are amplified for library construction and sequencing. **(C)** In 10x Genomics scATAC-seq, nuclei are transposed and encapsulated into droplets using the microfluidics chip. Next, the collected single nuclei GEMs are linearly amplified, and barcoded accessible DNA fragments are obtained after breaking the emulsion. Finally, DNA fragments are ready for library construction and sequencing. **(D)** In CyTOF, the cells are labeled using stable heavy metals, nebulized, and vaporized to form ion clouds through an argon plasma torch. Each cloud passes through a quadrupole which performs a purification step, the remaining heavy ions are quantified by a time-of-flight (TOF) mass spectrometer that determines the value of each marker. **(E)** In co-detection by indexing (CODEX), FFPE or FF tissues samples are stained with DNA-barcoded antibodies. Next, a multicycle reaction characterized by iteratively imaging up to three antibodies and nuclear stain, stripping and hybridizing is performed. This process is performed for all antibodies. Finally, raw images are processed and analyzed. **(F)** In CITE-seq, antibody-derived tags (ADTs) are used to bind the cells of interest. Next, cells are incapsulated into droplets using a microfluidics platform and after cell lysis in droplets, mRNAs and ADTs are barcoded during the RT. After amplification, cDNAs and ADTs are separated by size, converted into two independent libraries that are, finally, pooled, and sequenced.

Finally, high-throughput methods capable of processing thousands of single cells per experiment were introduced, such as those based on microfluidics and barcoding (SiC-seq) (Lan et al., 2017), split-pool strategies (SCI-seq) (Vitak et al., 2017), high-throughput versions of linear amplification via transposon insertion (sci-L3) (Yin et al., 2019), and direct library preparation (DLP+) (Laks et al., 2019). The aim of high-throughput methods was introducing automation to increase the number of cells analyzed while maintaining accuracy of genomic information. SiC-seq exploits droplet microfluidics to encapsulate cells into microspheres in which to perform the reactions required for cell and genome processing without compromising genomic DNA. SiC-seq performs a series of steps to lyse the cells, fragment the genomes, barcode the DNA fragments and sequence them after library preparation. SCI-seq uses the strategy of transposase-based combinatorial indexing (Adey et al., 2010) to obtain barcoded libraries without using droplet microfluidics. Sci-L3 addresses the problem of genomic artifacts due to PCR amplification and low-throughput using respectively linear amplification and a three-level combinatorial indexing. DLP + takes advantage of specialized hardware and software for imaging microscopy to capture genomic information of thousands of cells per experiment. Given the complexity in obtaining single cell genomic information, commercial solutions such as those based on the 10x Genomics droplet microfluidics system have been also proposed to simplify single-cell genomics data generation (Figure 2A). Single-cell genomic technologies have been used, for example, for the deconvolution of clonal cell clusters and tracing the evolutionary trajectories of clonal breast cancer cells (Wang et al., 2014), the identification of structural and mutational events of melanoma cell line clones (Velazquez-Villarreal et al., 2020), and for an in-depth view of the intratumoral copy number alteration (CNA) heterogeneity present in breast cancer genomes (Baslan et al., 2020).

All these aspects offer a greater understanding of the dynamics of the TME, especially in the cancer therapy response context. For example, identifying clonal and sub-clonal cell composition or the presence of specific mutated subsets would be useful for stratifying patients to understand whether they could benefit from immunotherapy. In the future, longitudinal studies coupled with other single-cell omics could help to construct a more detailed map of clone evolution kinetics and elucidate the time-dependent mechanisms underlying therapy and the emergence of more aggressive clones caused by selection pressures (McGranahan and Swanton, 2015). Furthermore, these studies could also suggest evolutionary time points with a favorable TME to perform more effective therapeutic interventions. In the future, the widespread use of single-cell genomics methods will depend on improvements of the actual technical limitations. Current technologies are not optimal to study all the different genomic aberrations such as copy number variations (CNVs), small indels, single-nucleotide variations and structural variations (SVs) at the same time (Fan et al., 2021). Advancements on the current protocols and bioinformatics solutions will be critical for a wider adoption of single-cell genomics by the scientific community.

### Single-Cell Transcriptomics

Single-cell RNA-sequencing (scRNA-seq) is a key technique to explore the TME. scRNA-seq has expanded the scenarios opened by previous bulk RNA-seq technology to investigate the transcriptome of single cells inside a biological sample. Despite countless discoveries in the last decade due to the ability to sequence millions of RNA fragments from a “bulk” of cells, it is now mandatory to look at the transcription machinery of each single cell to understand the complexity of the TME. Indeed, the possibility of observing the expression of thousands of genes for each cell has allowed us to resolve cell subset heterogeneity of the TME in an unbiased way (Lambrechts et al., 2018; Zilionis et al., 2019). Furthermore, scientists can go beyond merely characterizing cell compositions and obtain information about cell state, differentiation trajectories, and cellular pathway activation (Qian et al., 2020; Zhang et al., 2020; Raghavan et al., 2021).

The TME is crucial for tumor cells to evade immune surveillance, necessitating detailed identities of cancer, immune, and non-immune cells. A central aspect in the fight against cancer requires an enhanced understanding of the role of immune cells in cancer therapies, especially in immunotherapy based on immune checkpoint inhibitors (ICIs). Hence, in recent years, scientists have tried to understand the reasons for the success or failure of immunotherapy in relation to the immune features of the TME. It is now clear that T cell infiltration into tumor tissues is a key feature of the immunotherapy response (Tang et al., 2016). However, the presence of T lymphocytes is a necessary but insufficient condition for an effective antitumor response. Indeed, the immunotherapeutic reactivity of a tumor largely depends on the functional state of infiltrated T cells and the expression of specific drug-targetable molecules, such as programmed cell death protein 1 (PD-1) and cytotoxic T lymphocyte-associated antigen 4 (CTLA-4), as well as and the lymphocyte-activation gene 3 (LAG-3), which negatively regulates T-cell proliferation and effector T-cell functions. Recently, a phase II/III, global, double-blind, randomized study named RELATIVITY-047, demonstrated that the dual inhibition of LAG-3 and PD-1, using relatlimab and nivolumab, had a synergistic effect on progression-free survival in melanoma patients highlighting the possibility to develop more effective therapy by targeting different T cell activation brakes (Tawbi et al., 2022). Furthermore, the expression of co-stimulatory molecules, such as programmed death-ligand 1 (PD-L1), by cancer cells and an inflamed TME are crucial elements for an effective immunotherapy response (Marigo et al., 2016; Duan et al., 2020; Ugel et al., 2021). In contrast, the presence of myeloid-infiltrating cells with immunosuppressive features, such as myeloid-derived suppressor cells (MDSCs) (De Sanctis et al., 2016) and a low frequency of infiltrating CD8^+^ T cells in the TME, correlate with tumors averse to immunotherapy (Zhu et al., 2017; Li J. et al., 2018).

Cancer immunology discoveries have continued in parallel with advancements in scRNA-seq technologies. Among others, methods based on droplet microfluidics and cellular barcoding, such as InDrop (Klein et al., 2015), Drop-seq (Macosko et al., 2015), and the 10x Genomics platform (Zheng et al., 2017) have gained attention in recent years. Similarly to single-cell genomics technologies, their success is due to the greater scalability offered by droplet microfluidics platforms that allow to isolate thousands of cells in a short time (Figure 2B). A drawback of these technologies is their low transcript coverage. Indeed, these methods cannot be used to study, for example, splicing and enhancer RNAs at the single-cell level (Hayashi et al., 2018) because they sequence only the 3’ end of each transcript. In contrast, full-length technologies, such as SMART-seq (Ramskold et al., 2012; Picelli et al., 2014) and MATQ-seq (Sheng et al., 2017), have a higher accuracy level in transcript detection, allowing the study of gene variants and splicing events at the cost of lower single-cell profiling throughput.

Given the complexity of the TME in terms of cellular heterogeneity, high-throughput scRNA-seq techniques have been widely used in cancer studies because they can extract thousands of cells from a biological sample. scRNA-seq has been successfully used to decipher several mechanisms by which the tumor can shape the microenvironment towards a hostile ecosystem characterized by exhausted infiltrating T cells, suppressive myeloid cell subsets, cells with pro-tumor differentiation programs, and aberrant cell-cell interaction networks (Guo et al., 2018; Elyada et al., 2019; Davidson et al., 2020; Kim et al., 2020; Marigo et al., 2020; Wu et al., 2021; De Sanctis et al., 2022). The integration of scRNA-seq data with clinical patient information has further allowed us to define cellular and molecular signatures of the TME to explain the response to immunotherapy or survival outcome in different types of cancer (Jerby-Arnon et al., 2018; Sade-Feldman et al., 2018; Ma et al., 2019; Peng et al., 2019; Di Pilato et al., 2021; Zhang et al., 2021). These results motivate future applications of scRNA-seq as a valuable tool for clinicians to perform TME screening, stratification, and identification of new druggable targets based on the integration of different patient datasets (Giladi and Amit, 2017; Binnewies et al., 2018). Data integration is of primary importance because transcriptional-level results cannot be directly translated to the functional level, requiring additional support and experimental validation at the protein level.

Recently, scRNA-seq has been combined with the innovative spatial transcriptomics (Zhang et al., 2022). This technology has revolutionized the traditional RNA-fluorescence *in situ* hybridization (FISH) tool able to identify target messenger RNA transcripts in tissue sections by-passing the absence of selective antibodies for unknown candidates (Facciponte et al., 2014; Cui et al., 2016). Indeed, this innovative approach allows to visualize profiles of RNA molecules in identified tissue regions, including technologies based on micro-dissected gene expression, *in situ* hybridization, *in situ* capturing and *in situ* sequencing technologies (Calvanese et al., 2022; Nirmal et al., 2022; Wei et al., 2022). This technology continues to aid the development of human cellular atlases of cancer, the reclassification of the immune landscape of TME, and overall, the identification of important therapeutic targets.

### Single-Cell Epigenomics

The epigenome is the complete atlas of chemical modifications that can induce changes in gene expression without modifying the DNA sequence. Such modifications involve DNA, RNA, and histone proteins and can cause chromatin remodeling that can turn genes “on” or “off”. Methylation and acetylation of histones on lysine and arginine residues are the best-known epigenetic mechanisms capable of enhancing or repressing gene transcription, as exemplified by histone H3 on lysine 27 (H3K27ac) and histone H3 on lysine 9 (H3K9me) modifications. Other epigenetic mechanisms that provoke chromatin remodeling include nucleosome positioning/reorganization and DNA methylation.

Several studies have highlighted the ability of tumor cells to induce epigenetic modifications in TME-infiltrating immune cells to aid immune surveillance evasion. The epigenetic strategies implemented by tumors to avoid immune surveillance are based on the disruption of different anti-cancer immune mechanisms, such as immune recognition, signal activation, and effector functions. Common mechanisms exploited by tumors to evade immune recognition and signal triggering include the epigenetic silencing of major histocompatibility complex (MHC) genes and the inhibition of cytokines and chemokines. For example, trimethylation of H3K4 (H3K4me3) and H3K27 (H3K27me3) by polycomb repressive complex 2 (PRC2) has been linked to MHC-I repression and missed tumor recognition by CD8^+^ T cells mediated by EED and EZH2 (Burr et al., 2019). Furthermore, the lack of tumor-infiltrating lymphocytes (TILs) in several human cancers is associated with DNA methylation-induced epigenetic silencing of *CCL5* (Dangaj et al., 2019). An illuminating example of how epigenetic modifications can result in the loss of immune effector function is the acetylation of H3K27 (H3K27ac). Indeed, high H3K27ac levels have been linked to a TNF-NFKB1 pathway capable of inducing CD47 upregulation and inhibiting macrophage phagocytosis of breast cancer cells (Betancur et al., 2017).

Our understanding of the cancer epigenome has evolved rapidly with the adoption of next-generation sequencing (NGS). In this context, chromatin immunoprecipitation followed by sequencing (ChIP-seq) and an assay for transposase-accessible chromatin using sequencing (ATAC-seq) have been widely used tools to study epigenetic regulation. ChIP-seq is a method for studying the interactions between proteins and DNA. It allows us to analyze chromatin states induced by histone modifications that alter gene transcription. ATAC-seq measures chromatin accessibility by directly deciphering its effects on gene transcription without detailed histone modification and chromatin state characterization. ChIP-seq and ATAC-seq have been used successfully, for example, to link epigenetic features capable of maintaining an immune cell-excluded TME and immunotherapy resistance (Benci et al., 2016; Yang et al., 2021).

The advancements of these techniques are their single-cell counterparts, scChIP-seq and scATAC-seq. Several cancer studies have shown the utility of scChIP-seq or scATAC-seq to study the epigenetic regulators responsible for tumor cell heterogeneity (Grosselin et al., 2019; LaFave et al., 2020; Taavitsainen et al., 2021). Other applications of single-cell epigenomics include adopting scATAC-seq to uncover chromatin regulators responsible for T cell exhaustion in the TME of patients treated with immunotherapy (Satpathy et al., 2019; Zhang et al., 2021). These examples motivate the use of single-cell technologies to study cancer epigenetics.

In the future, it will be pertinent to understand the link between epigenetic changes in the TME and cancer progression and how to obtain a more effective therapy response targeting epigenetic switches. One reason for the growing interest in targeting the epigenome is the possibility of identifying small molecules, such as proteolysis-targeting chimeras (PROTAC) (Sakamoto et al., 2001), that can indirectly interfere with aberrant gene expression, given the difficulties in targeting oncogenic transcription factors, such as Myc and p53 (Jones et al., 2016).

The dissemination of scChIP-seq studies depends on future technological advances. Despite recent successful attempts to improve scChIP-seq in terms of cellular sensitivity and data sparsity (Kaya-Okur et al., 2019; Wang et al., 2019), the use of this technology remains limited and largely relies on being able to increase the number of sequenced reads per cell. In contrast, scATAC-seq has a simpler and more efficient experimental protocol that requires a lower number of cells. Furthermore, introducing microfluidics approaches (Figure 2C) has dramatically improved the throughput compared to the previous technology based on combinatorial cellular indexing (sciATAC-seq) (Cusanovich et al., 2015). However, low per-cell coverage remains a weakness, even for scATAC-seq, potentially limiting the identification of significant open chromatin sites, especially in rare cell subsets (Ma and Zhang, 2020).

### Single-Cell Proteomics

Despite the wide adoption of single-cell transcriptomics technologies, single-cell proteomic approaches remain the key tools for studying the functional status of TME cell populations. Indeed, in addition to identifying proteins and related isoforms, they allow us to recognize post-translational modifications that single-cell transcriptomics cannot capture.

FC is still a fundamental multiparametric technique for identifying immune cell subsets within the TME based on morphological characteristics and the expression of certain proteins. FC also allows specific cell populations to be isolated by cell sorting before analysis with other omics techniques. The main limitation of FC is the number of parameters, usually around 20, which can be intercepted simultaneously in the same experiment because of signal overlap (i.e., spillover) between the channels. This limits the number of cell-surface proteins that can be identified and, consequently, the resolution of identifying the related cell subsets present in the experiment.

A more advanced technology is Full Spectrum Flow Cytometry (FSFC), an improvement of spectral flow cytometry (SFC) (Robinson, 2004; Nolan et al., 2013). FSFC exploits high-sensitive light detectors to measure the full spectral profile of fluorophores. This technology has a higher quality and resolution than conventional FC, allowing to design multicolor panels up to 40 parameters useful for characterizing important aspects of the immune context in cancer studies (Bonilla et al., 2020).

Another recent technology for single-cell proteomics is cytometry by time-of-flight (CyTOF) (Bandura et al., 2009) (Figure 2D). Compared to FC, CyTOF allows at least 40 markers per cell to be detected in a single run and is more sensitive and less prone to errors (Bandura et al., 2009; Bendall et al., 2011). The disadvantages of CyTOF compared to FC include a lower acquisition flow rate, more critical sample preparation to avoid contamination, and the inability to perform cell sorting for populations of interest due to the final vaporization of the cells (Gadalla et al., 2019). CyTOF potentiates better resolution of TME heterogeneity, which is particularly important in the context of cancer immunology. For example, CyTOF makes finding specific TIL subsets that correlate with patient survival and response to immunotherapy, respectively, in follicular lymphoma (Yang et al., 2020) and melanoma (Subrahmanyam et al., 2018) possible. Furthermore, scientists were able to localize the expression of the T cell inhibitory molecule VISTA in CD68^+^ macrophages of human pancreatic cancer (Blando et al., 2019) and retrieve information on the composition, expansion, and activity of TILs in patients with non-small cell lung cancer (NSCLC) (Sanmamed et al., 2021). Since CyTOF dissects the cellular composition and the activation status of the immune cells that surround and infiltrate the tumor, it is an effective tool for studying the TME immune landscape. CyTOF and scRNA-seq allowed us to answer similar biological questions. However, these two technologies are not interchangeable. The main limitations of CyTOF are the resolution and bias due to prior parameter selection. These two aspects can limit the discovery of rare cell populations. An obvious advantage of CyTOF is that protein expression indicates specific functional states or activities of the cell that in scRNA-seq must be validated with other techniques. Since CyTOF is simpler than scRNA-seq and allows for better discrimination of certain immune cell subsets (Kashima et al., 2021), it represents a convenient method to monitor TME features, such as cell subsets, activation states, and immune checkpoint molecules in patient cohorts in clinical trials. It is mandatory to rapidly standardize experimental procedures, computational tools, and antibody panels to make results comparable between different institutions to accomplish this aim (Hartmann et al., 2019).

In CyTOF, protein identification strictly depends on the availability of highly specific antibodies and the quality of their interactions. Mass spectrometry-based methods, such as SCoPE-MS and the improved version SCoPE2, have been recently introduced to improve throughput and sensitivity concerning the number of proteins detected in single cells (Budnik et al., 2018; Specht et al., 2021). With SCoPE2, the authors were able to dissect cellular heterogeneity by protein expression and trace the differentiation of monocytes into macrophage-like cells in the absence of specific cytokines. This work is an important step towards future applications of single-cell technologies based on mass spectrometry for TME dissection, looking at the expression of thousands of proteins.

### Single-Cell Spatial Proteomics

An important aspect to consider in the study of the TME is its spatial organization and heterogeneity. Indeed, like natural ecosystems, tumor tissues can reveal strong heterogeneity in relatively small spatial distances due to tumor cells adapting to the microenvironment or through its remodeling (Yuan, 2016). Several studies have emphasized the importance of considering spatial heterogeneity in the TME. Some of these reports have shown that the location, density, and spatial distribution of immune cells are more robust markers for predicting patient outcomes than traditional clinical parameters (Galon et al., 2006; Maley et al., 2015). These facts motivated the development and improvement of technological platforms for spatial analysis and their use in dissecting the TME.

IHC is a widely used technique for both basic research and cancer diagnosis. It is used to localize cells that express specific protein markers and study the spatial localization of cells in a tissue slide. In conventional IHC (i.e., chromogenic IHC), antibodies recognize specific antigens in the tissue and are conjugated to an enzyme to catalyze a color-producing reaction. A major problem with conventional IHC is that it only allows labeling of one marker for tissue sections. This severely limits understanding the cellular complexity of the TME. Several multiplex platforms have been introduced over the years to facilitate the analysis of cellular composition, functional states, and cell-cell interactions within the TME to address this problem. Multiplex IHC (mIHC) methods based on chromogens (Remark et al., 2016; Tsujikawa et al., 2017), fluorophores (Gerdes et al., 2013; Gorris et al., 2018; Viratham Pulsawatdi et al., 2020), metal-tagged antibodies (Angelo et al., 2014), and DNA barcodes (Goltsev et al., 2018; Manesse et al., 2020) (Figure 2E) increased the number of biomarkers to be used simultaneously by up to 50 in a single tissue section. The introduction of more effective mIHC technologies has been accompanied by the development of new software solutions capable of performing sophisticated analyses of digitalized images (e.g., segmentation and filtering) and support the work of pathologists in sample processing.

mIHC technologies have been used in different types of cancer to better understand the spatial architecture of the TME and how it can affect the response to therapy and clinical outcomes. For example, in breast cancer, mIHC was used to obtain detailed information on the spatial localization, cellular composition, and expression of regulatory proteins in the TME and recover clinically relevant characteristics (Keren et al., 2018; Jackson et al., 2020). In colorectal cancer, mIHC has been used to decipher the complex dynamic interplay between TME components (Schurch et al., 2020). In human pancreatic ductal adenocarcinoma (PDAC), mIHC was applied to assess the density and spatial distribution of myeloid and lymphoid cells in the TME and its correlation with the clinical outcome of patients (Liudahl et al., 2021).

### Single-Cell Multimodal Omics

In cancer, single-cell omics aims to dissect all aspects of cellular machinery to understand its functional status and relationship with other TME cells. The use of single-cell omics technologies to study single-cell modalities, such as transcriptomics and proteomics, has increased our knowledge of cell biology in cancer without precedent. However, cells are dynamic entities whose states are characterized by a complex interplay of genomic, transcriptomic, epigenomic, and proteomic features that non-linearly contribute to the TME’s heterogeneity. The new Frontier is the simultaneous measurement of multiple modalities of the same cells to gain a better understanding of cellular and molecular mechanisms in cancer. In recent years, this has motivated the introduction of numerous single-cell approaches capable of combining two or more modalities between genomics, transcriptomics, epigenomics, and proteomics (Figure 1E). Multimodal single-cell approaches have evolved from those using tubes or microwells of plates to measure single cells (i.e., low-throughput) to those that take advantage of droplet-based technologies or combinatorial DNA barcoding strategies (i.e., high-throughput), allowing increased scalability and reduced costs per run (Zhu et al., 2020). Almost all current single-cell multimodal omics technologies extract the transcriptome of each cell. Low-throughput multimodal omics with transcriptomics paired with genomics are gDNA-mRNA sequencing (DR-seq) (Dey et al., 2015) and genome and transcriptome sequencing (G&T-seq) (Macaulay et al., 2015), while transcriptomics and epigenomics are obtained with single-cell methylome and transcriptome sequencing (scM&T) (Angermueller et al., 2016), scMT-seq (Hu et al., 2016), single-cell nucleosome, methylation, and transcription sequencing (snNMT-seq) (Clark et al., 2018), single-cell nucleosome occupancy, methylome, and RNA expression sequencing (scNOMeRe-seq) (Wang et al., 2021), and single-cell chromatin accessibility and transcriptome sequencing (scCAT-seq) (Liu et al., 2019). An interesting multimodal omics technique integrating transcriptomics, genomics, and epigenomics is single-cell triple omics sequencing (scTrio-seq) (Hou et al., 2016).

In high-throughput multimodal omics, transcriptomics is paired with epigenomics in parallel analysis of individual cells for RNA expression and DNA accessibility by sequencing (Paired-seq) (Zhu et al., 2019) and single-nucleus chromatin accessibility and mRNA expression sequencing (SNARE-seq) (Chen S. et al., 2019). Furthermore, transcriptomics is paired with epitopes in RNA expression and protein sequencing (REAP-seq) (Peterson et al., 2017) and cellular indexing of transcriptomes and epitopes by sequencing (CITE-seq) (Stoeckius et al., 2017) (Figure 2F). CITE-seq protocol has been extended to obtain transcriptomics and proteomics of the same cells after CRISPR-Cas9 genetic perturbations in expanded CRISPR-compatible CITE-seq (ECCITE-seq) (Mimitou et al., 2019) and Perturb-CITE-sequencing (Perturb-CITE-seq) (Frangieh et al., 2021). These techniques perform genetic perturbations through single gRNAs (sgRNA) linking them to transcriptomic and proteomic profiles of the same cells to allow demultiplexing. ECCITE-seq combines the use of CRISPR libraries and cell hashtags (Stoeckius et al., 2018) to perform genetic perturbations and pool together different experimental samples. Perturb-CITE-seq uses a method based on CROP-seq (Datlinger et al., 2017) to express sgRNAs and to link them to transcripts and surface proteins of single cells. Other interesting multimodal omics techniques that do not include transcriptomics are, for example, those that provide different layers of epigenomics (Guo et al., 2017; Pott, 2017), epigenomics paired with genomics, and CRISPR-Cas9 genetic perturbations (Rubin et al., 2019; Tedesco et al., 2021).

Low-throughput multimodal omics were successfully adopted in cancer studies to better understand heterogeneity, complexity, and the evolution of cancer cells by integrating genomic, epigenomic, and transcriptomic features of the same cells (Macaulay et al., 2015; Hou et al., 2016; Bian et al., 2018; Zhu et al., 2021). However, the possibility of obtaining only tens or hundreds of cells provided by low-throughput multimodal omics and their costs have limited their application in TME studies.

Furthermore, given the importance of immunomodulatory proteins (e.g., PD-L1, CTLA-4) in response to cancer immunotherapy, high-throughput multimodal technologies combining transcriptomics and proteomics have been applied in TME studies. In a recent report, CITE-seq successfully discovered new macrophage populations expressing PD-L1 and PD-L2 surface proteins linked to survival in breast cancer (Wu et al., 2021). In other studies, ECCITE-seq and Perturb-CITE-seq were used to define new clinically relevant resistance mechanisms to ICIs in human cancer cell lines and melanoma by exploiting CRISPR-Cas9 screens with multimodal single-cell readouts (Frangieh et al., 2021; Papalexi et al., 2021). Furthermore, the possibility of having both transcript and protein expression from the same cells also helped scientists increase the robustness of their scRNA-seq results. Indeed, they were able to validate the expression of known protein markers and identify novel proteins expressed by immune subsets in different human cancers (Leader et al., 2021; Pombo Antunes et al., 2021). The progress in single cell technologies will guarantee in the near future the possibility to explore at single cell level other omics such as glycomics, lipidomics, metabolomics and microbiomics able to highlight essential cell functions and biological proprieties of tissue components generating a more detailed map of the immune landscape of TME.

## Computational Approaches to Analyze Single-Cell Data of the TME

With the widespread use of high-dimensional biological datasets, the scientific community designed specific computational techniques capable of extracting knowledge from complex multi-omic data. Compared to its bulk counterpart, single-cell data analysis is particularly challenging because of the high dimensionality given by the number of cells and markers and the presence of peculiar technical and biological factors that are important to keep in mind. For example, an underestimated problem in single-cell analysis is computational power. As the number of cells increases, all the data analysis steps become more computationally intensive, and some of these steps may require more scalable computational methods and architectures. Machine learning (ML) techniques, especially deep learning (DL), are an emerging class computational methods in single-cell data analysis for their capability to manage complex datasets and the possibility to be implemented into high-parallel architectures (e.g., GPU). Additionally, single-cell data is noisier and more subject to batch effects compared to the bulk counterpart due, for example, to more critical experimental procedures such as single cell isolation, the technical variability in the number of reads and cells sequenced in each sample, and the biological variability caused by heterogeneity in cell composition. TME studies particularly exemplify the latter aspect, in which the presence of cancer and immune and non-immune cells contributes to the complexity of the experiment. Potential confounding factors must be removed or included in statistical models during data analysis. Typical computational steps performed during single-cell data analysis include 1) preprocessing and harmonization steps in which multiple datasets or modalities are combined to perform an integrated analysis after the removal of outliers or low-quality cells, 2) applying dimensionality reducing techniques useful for visualization, 3) clustering, 4) cell annotation, and 5) cellular and molecular functional analysis.

### Pre-Processing and Harmonization of Single-Cell Data

Pre-processing is essential before the downstream analysis of single-cell data. This phase encompasses many computational steps ranging from raw to processed data through various types and file formats. Sequencing- and mass spectrometry-based single-cell data involve several steps before quantifying the features-by-cell matrices. Sequencing-based methods have common procedures, including processing raw FASTQ files containing the reads and alignments to the reference genome or transcriptome. Next, for both sequencing-based and mass spectrometry-based approaches, feature detection and quantification (e.g., exons, peaks, and peptides) are common stages to obtain the final features for analysis. Prior to matrix processing and analysis, preliminary quality control is required to assess, for example, the quality of the reads, the percentage of valid barcodes, and reads mapped to the genome. The software suite of specific platforms (e.g., 10x Genomics Cell Ranger) often provides this information. Next, the matrices are reduced by removing low-quality and outlier cells. For example, cells with an unexpectedly high or low number of features detected or with poor quantification are removed from further analyses. In droplet-based single-cell technologies, a filtering step removes cells with a hybrid transcriptome (e.g., doublets), that is, two or more cells incapsulated in the same droplet, using specialized software (McGinnis et al., 2019; Wolock et al., 2019; DePasquale et al., 2020). A specific scRNA-seq pre-processing step involves removing cells with a high percentage of mitochondrial and ribosomal genes expressed because they are usually considered low quality. Thresholds on mitochondrial and ribosomal expression must be chosen carefully, especially when dissecting the immune complexity of the TME, to avoid removing certain cell subsets (Zilionis et al., 2019; Osorio and Cai, 2021; Subramanian et al., 2021).

In FC and CyTOF, pre-processing steps and downstream analyses are typically performed using flow cytometry standard (FCS) files. Typical FC pre-processing steps include data compensation and transformation (e.g., biexponential, generalized Box-Cox) to correct the channel spillover and the effects of outliers and distorted distributions, respectively. Like droplet-based single-cell technologies, an important FC pre-processing step involves removing doublets. This happens when the cytometer cannot discriminate between 2 cells because they pass too closely through the trigger laser. In FC, single cells are differentiated at the beginning of the gating strategy using the 2D plot with Forward Side Channel-Aria (FSC-A) and Forward Side Channel-Height (FSC-H). Cells that do not display a linear correlation of these two parameters are marked as doublets and are excluded from the analysis. In CyTOF, the discrimination of doublets is more complex because the cell size parameters used in FC are not available. Here, DNA intercalators and event length are used to obtain single cells (Gadalla et al., 2019). In spatial proteomics, the pre-processing step involves some adjustment (e.g., illumination and contrast correction) to the images before cell identification through the segmentation process. After segmentation, the cells are classified and quantified for subsequent statistical analysis.

As part of the pre-processing phase, normalization is a fundamental procedure in single-cell data analysis to make all the cells comparable. In sequencing-based single-cell data, the variability of reads sequenced per cell and data sparsity have been carefully considered in computational pipelines to avoid technical effects confounding biological heterogeneity. In scRNA-seq, this has motivated the design of different normalization methods, for example, those based on the estimation of size factors to correct gene expression (Lun et al., 2016; Wolf et al., 2018; Stuart et al., 2019) and others based on linear regression (Bacher et al., 2017; Yip et al., 2017; Hafemeister and Satija, 2019). Text-mining techniques are used to normalize scATAC-seq datasets (Cusanovich et al., 2015; Cusanovich et al., 2018; Fang et al., 2021). Normalization is also an important step in single-cell proteomics data to remove the technical variability due, for example, to differences in instrument performance over acquisition time, particularly in mass cytometry (Rybakowska et al., 2020). Bead-based normalization was introduced to correct these technical artifacts (Finck et al., 2013). This method uses information obtained through standard calibration beads that track changes in the signal over the acquisition time to adjust the marker values. However, technical and biological differences may arise from the disparate technical and biological aspects of data generation in single-cell experiments. A key step in studying the TME through single-cell technologies is the study of changes in the cell subset composition of different individuals. Harmonization techniques have been proposed for single-cell data to facilitate the comparison of multiple samples. These techniques aim to overcome noise due to the variability among cells, individuals, species, and protocols trying to maintain the true biological signals. An important advantage of data harmonization is the possibility to aggregate multiple samples into a single dataset making faster identifying shared or sample-specific cell subsets. This approach makes the analysis simpler and less error prone because it avoids processing each dataset individually or merging them without considering potential biases. A drawback of harmonization is the possibility to lose true biological signals due to the “correction” procedure. In single-cell proteomics, methods have been introduced to correct unwanted variability in FC and mass cytometry, such as aligning the marker intensity distributions across samples or performing cell-type-specific normalization using shared controls across multiple batches (Hahne et al., 2009; Finak et al., 2014; Van Gassen et al., 2020). Several methods ranging from more conventional methods to highly scalable and fast ML approaches have been proposed to align and make cell subsets comparable to gene expression and chromatin accessibility of different datasets, considering diverse technical and biological variation sources (Luecken et al., 2022). The advent of single-cell multimodal omics has posed additional computational challenges in extrapolating useful information from the different layers measured for each cell. This has motivated the introduction of harmonization methods for performing a joint analysis, such as clustering, to exploit the power given by all available cell modalities (Argelaguet et al., 2020; Wang et al., 2020; Gayoso et al., 2021; Hao et al., 2021; Singh et al., 2021; Zuo and Chen, 2021). It is worth to remark that DL approaches (e.g., deep generative models) represent a significant part of harmonization methods for single-cell datasets. Their power is given by their capacity to learn complex mechanisms of biological systems from multiple biological datasets and modalities (Li Y. et al., 2018). This makes them important tools to manage the increasing complexity of single-cell data.

### Dimensionality Reduction, Clustering, and Cell Annotation of Single-Cell Data

Single-cell datasets are intrinsically high dimensional. A common step in single-cell data analysis is projecting the data into a low-dimensional space using dimensionality reducing techniques to dissect the complexity and understand the TME’s cellular composition, cell states, and trajectories. The dimensions calculated by these algorithms are useful for visualizing the cell subsets, usually using 2D scatter plots, and as inputs of other computational techniques. Methods such as principal component analysis (PCA) and singular value decomposition (SVD) are commonly used to decompose high-dimensional datasets into several important axes of variation. Owing to their linearity, these methods cannot accurately represent the complex structure of single-cell data. However, the dimensions extracted by these approaches are commonly injected into other dimensionality reducing methods, such as t-distributed stochastic neighbor embedding (t-SNE) and uniform manifold approximation and projection (UMAP), to improve their accuracy (Kobak and Berens, 2019; Baek and Lee, 2020; Kobak and Linderman, 2021). t-SNE and UMAP are currently the most commonly used techniques for visualizing single-cell datasets intuitively. With these methods, each cell is represented as a point in a 2D scatter plot, where the proximity to other cells indicates a similarity in expression profiles. These algorithms can position cells in a biologically meaningful way allowing the correct interpretation of the data. A vital property of these algorithms is to preserve the local and global structure of the data, namely, the distances between points (e.g., cells) within the same cluster and between different clusters. The local topology ensures that the cells in a cluster are homogeneous and represent, for example, the same immune cell subset. In contrast, the global structure of data can provide important biological insights into cell subset relationships, such as monocyte-to-macrophage or epithelial-to-malignant cell transitions, and it is the most debated feature of t-SNE and UMAP. Although previous reports have shown UMAP to be better than t-SNE in preserving the organization of cell clusters (Becht et al., 2018), recent studies suggest that more effective t-SNE parameterizations make it as good as UMAP for conserving global data geometry (Kobak and Berens, 2019; Kobak and Linderman, 2021). Considering the importance of studying the state transition and differentiation trajectories of single cells, other dimensionality reducing approaches have been proposed to better preserve the global features of the data and, consequently, provide biologically relevant information (Weinreb et al., 2018; Moon et al., 2019). After dimensionality reduction, clustering and cell annotation are usually the next steps in single-cell data analysis. Clustering algorithms are mainly used to define homogenous cell subsets to be annotated using specific molecular measures, such as gene expression, proteins, or peak counts. In single-cell multimodal omics data, all the provided molecular measures can be combined to perform an integrated analysis, as the previous section introduced.

Among the large toolboxes of clustering techniques made available by computational sciences, several methods are more commonly used in the single-cell data analysis context. Graph-based clustering has become very popular in single-cell data analysis owing to its minimal required assumptions compared to other well-known techniques, such as k-means. These include methods based on clique detection (Xu and Su, 2015), spectral clustering (Ng et al., 2002), and community detection algorithms (Blondel et al., 2008; Waltman and Van Eck, 2013; Traag et al., 2019). The latter has been highly appreciated in recent years because they can scale the number of cells and be implemented in popular R and Python packages, such as Seurat (Stuart et al., 2019), Monocle (Qiu et al., 2017), and SCANPY (Wolf et al., 2018). In recent years, ML techniques based on self-organizing maps (SOMs) (Kohonen, 1990) have also been introduced for both mass cytometry (Van Gassen et al., 2015) and scRNA-seq data (Camp et al., 2017). These tools usually provide useful visualization features for an intuitive interpretation of expression similarity among cell clusters.

Cluster analysis is usually used for cell annotation following an iterative process in which each cluster is mapped to a biologically relevant cell type by observing the expression of multiple markers. Several clustering algorithms allow us to set a level of granularity (i.e., resolution) based on the level of detail at which the cell subsets are dissected. The use of clustering to annotate cells has several drawbacks. For example, the number of clusters may be overestimated, underestimated, or not reproducible. Furthermore, manual annotation may be error-prone or not correspond to similar annotated cells in the literature. Alternatively, several classification techniques have been introduced in recent years to perform automatic cell annotation (Zhao X. et al., 2020) based on well-annotated reference datasets containing, for example, fluorescence-activated cell sorting (FACS)-sorted cell populations. These methods typically use bulk or single-cell references from large general-purpose (Consortium, 2012; Mabbott et al., 2013; Martens and Stunnenberg, 2013), immune-specific (Heng et al., 2008), or tumor-specific atlases (Rozenblatt-Rosen et al., 2020) to infer the cell types present in dataset by correlating their expression profiles. Most of these tools make feature selections before performing cell classifications. However, the unbiased nature of these approaches makes them critical for improving the reproducibility and consistency among single-cell studies. With the spread of large-scale disease datasets, an important goal will be to integrate these datasets into harmonized and batch-corrected references to be queried efficiently. Scalable architectures based on ML will be valuable tools for integrating human references to study the TME (Lotfollahi et al., 2022).

### Analysis of Cell Function and Differentiation of Single-Cell Data

Analyzing changes in the cellular composition of the TME is necessary for understanding the various mechanisms of cancer. However, a deep molecular characterization is crucial for identifying the key drivers of functional cellular changes. Differential expression (DE) analysis is the main statistical technique for detecting functional perturbations caused by changes in gene or protein expression, chromatin accessibility, and genomic aberrations. Although general-purpose statistical tests or DE methods for bulk datasets have been widely used for DE analysis of different single-cell omics datasets, several specialized techniques have been adopted over the years to deal with the heterogeneity and sparsity of scRNA-seq (Kharchenko et al., 2014; Finak et al., 2015). However, DE methods based on pseudo-bulk aggregation of biological replicates have recently gained attention for their capacity to extrapolate more robust results than general-purpose and specialized single-cell DE methods (Squair et al., 2021). After differential expression analysis, a typical task in the study of the TME is to extract a list of biological processes linked to the molecular changes induced by cancer or therapy in different cell subsets. Methods for gene set analysis that have been widely used in recent years for bulk data, such as over-representation, are commonly used in single-cell data. These approaches take into account a list of differentially expressed molecules and gene sets from Gene Ontology (GO) (Gene Ontology, 2015), the Kyoto Encyclopedia of Genes and Genomes (KEGG) (Kanehisa et al., 2016), Reactome (Fabregat et al., 2018), and the Molecular Signature Database (MSigDB) (Liberzon et al., 2011) to identify altered molecular pathways.

In addition to molecular changes concerning pathway regulation, characterizing molecular states between different cells is a common step in single-cell data analysis. While clustering and cell annotation techniques provide information about the cell types present in the experiment, they supply no information on the relationships between cell types and clusters. Recently, several methods have been proposed to reconstruct differentiation trajectory maps by ordering cells based on expression pattern similarity (Saelens et al., 2019) or transcriptional dynamics (La Manno et al., 2018; Bergen et al., 2020). These techniques have been successfully applied to study the developmental trajectories of different cell types and TMEs (Zhang et al., 2019; Chen et al., 2020; Qian et al., 2020; He et al., 2021; Liu et al., 2021). With the improvement in single-cell omics technology performance and their integration ability, we need to address the problem of cell number scalability and the generalizability of these methods to different omics (Stassen et al., 2021).

Pro- and anti-tumor mechanisms are governed by changes driven by complex molecular and cellular interactions within the TME (Anderson and Simon, 2020). Various methods have been introduced in single-cell data analyses to study the intra- and inter-cellular interactome through the inference of gene regulatory networks (GRNs) and ligand-receptor pairs to understand the biological processes underlying these mechanisms. Several methods for GRN inference have been proposed to decipher intracellular networks, including tools originally designed for bulk transcriptomics (Huynh-Thu and Sanguinetti, 2015; Moerman et al., 2019) and techniques specifically designed for single-cell transcriptomics that exploit additional information, such as pseudo-temporal ordering (Matsumoto et al., 2017; Specht and Li, 2017; Deshpande et al., 2021) and information about transcription factors and their targets (Aibar et al., 2017). The first class of methods tries to learn the gene regulatory structure without prior information, and the second class uses pseudo-temporal information to better explain gene regulation during cell differentiation and development. A systematic evaluation of these techniques was recently published (Pratapa et al., 2020). In addition to the intracellular regulation state of a cell, a crucial aspect in understanding the TME involves exploring the cell-to-cell interactome induced by cancer cells (Whiteside, 2008). In spatial technologies, this task can be accomplished by observing cellular co-localization by inspecting appropriate cell markers in histological regions of interest (ROIs) of tissue sections. Without spatial information, cellular interactions must be inferred from the ligand and respective cognate receptor expression levels.

Several computational methods based on different mathematical models have been proposed to identify cell-to-cell interactions. Among others, methods based on the permutation of expression have been widely used (Armingol et al., 2021). These methods typically calculate the communication score of a list of ligand-receptor pairs obtained from curated databases and evaluate the significance of the interactions through cluster label permutation and statistical tests. Ligand-receptor interactions inferred from single-cell transcriptomics may provide interesting hypotheses that need to be further validated using other technologies.

## Conclusion and Future Perspectives

The shift from bulk to single-cell sequencing has allowed us to move forward from a general molecular signature in which the contribution of each cell is averaged to the complete molecular fingerprint of each sequenced cell. This is particularly important when complex samples characterized by heterogeneous cell compositions, such as tumor tissues, are analyzed. Accordingly, the employment of single-cell technologies has radically improved the understanding of the cancer framework both quantitatively and qualitatively. Indeed, a single-cell platform can resolve the plasticity of tumor cells and decode tumor phenotypes (invasiveness, stemness, proliferation, and apoptosis), revealing the composition of the TME and the differentiation of immune and stromal cells towards anti- or pro-tumor phenotypes. In addition, single-cell sequencing can track the evolutionary trajectories of neoplastic clones in primary tumors, with results that challenge the original vision of gradual neoplastic evolution (Gao et al., 2016) and improve the understanding of the metastatic spreading process by profiling circulating tumor cells or metastatic lesions (Leung et al., 2017). Similarly, molecular tumor fingerprinting can predict the response to target therapy (Tirosh et al., 2016; Rambow et al., 2018). Although single-cell technologies were initially developed for research purposes and contributed significantly to dissecting cancer evolution mechanisms, clinical settings will soon use them. Investigation at the single-cell level can improve early tumor detection, prognostic biomarker identification, and patient risk stratification, thus supporting a more tumor-tailored therapy. Similarly, dissecting the complexity of the TME can help design the best immunotherapy approach, reverting local immune suppression or empowering the fitness and killing abilities of tumor-infiltrating effector cells. Finally, single-cell sequencing platforms can be employed as potent diagnostic tools for non-invasive monitoring of tumor evolution and patient relapse by profiling circulating tumor cells. This approach can detect, for instance, the appearance of clones resistant to targeted therapy, promptly driving the clinical decision towards an alternative therapeutic solution. However, some limitations need to be overcome to make this technology available for mainstream clinical purposes. First, its usage requires a more complex team, including surgeons, oncologists, pathologists, and researchers working in a fast and coordinated manner and the development of robust tissue processing protocols for primary tumors. Moreover, sample processing requires loss of tissue architecture, whereas spatial single-cell technologies combining molecular and histological information do not guarantee the same resolution of single-cell sequencing on suspension cells. Secondly, scRNA-seq data depend on their intrinsic noisy since eukaryotic transcription does not occur at a persistent basal rate but it takes place in pulses (Chubb et al., 2006). Therefore, a failure to uncover a transcript of a specific gene in a cell at a single time point is an ambiguous result since it can be considered as a result of either permanent gene inactivation or timely limitation of gene transcription detection where the gene is active but the transcript in the time window of the sampling is not present. To avoid possible serious faults, the interpretation of scRNA-seq results should directed on pathways analysis and gene-set enrichment rather than single gene expression. Finally, batch effects can take places when aggregating multiple samples. Several methods of correcting batch effects have been optimized but their use must be balanced against the risk of eclipsing true biological differences. Nonetheless, as recently exemplified by the introduction of bulk NGS, single-cell technology use will soon be extended to patients to support cancer diagnosis and treatment in the near future.
